# Long-term ecology resolves the timing, region of origin and process of establishment for a disputed alien tree

**DOI:** 10.1093/aobpla/plv104

**Published:** 2015-08-26

**Authors:** Janet M. Wilmshurst, Matt S. McGlone, Chris S.M. Turney

**Affiliations:** 1 Landcare Research, PO Box 69040, Lincoln 7640, New Zealand; 2 School of Environment, The University of Auckland, Private Bag 92019, Auckland 1142, New Zealand; 3 School of Biological, Earth and Environmental Sciences, University of New South Wales, NSW 2052, Australia

**Keywords:** Alien, Asteraceae, dispersal, facilitation, historical ecology, invasion, *Olearia lyallii*, palaeoecology, pollen, subantarctic islands

## Abstract

Alien plants are a pervasive environmental problem, particularly on islands where they can rapidly transform unique indigenous ecosystems. However, often it is difficult to confidently determine whether a species is native or alien, especially if establishment occurred before historical records. This can present a management challenge: for example, should such taxa be eradicated or left alone until their region of origin and status are clarified? Here we show how combining palaeoecological and historical records can help resolve such dilemmas, using the tree daisy *Olearia lyallii* on the remote New Zealand subantarctic Auckland Islands as a case study. The status of this tree as native or introduced has remained uncertain for the 175 years since it was first discovered on the Auckland Islands, and its appropriate management is debated. Elsewhere, *O. lyallii* has a highly restricted distribution on small sea bird-rich islands within a 2° latitudinal band south of mainland New Zealand. Analysis of palaeoecological and historical records from the Auckland Islands suggest that *O. lyallii* established there c. 1807 when these islands were first exploited by European sealers. Establishment was facilitated by anthropogenic burning and clearing and its subsequent spread has been slow, limited in distribution and probably human-assisted. *Olearia lyallii* has succeeded mostly in highly disturbed sites which are also nutrient enriched from nesting sea birds, seals and sea spray. This marine subsidy has fuelled the rapid growth of *O. lyallii* and allowed this tree to be competitive against the maritime communities it has replaced. Although endemic to the New Zealand region, our evidence suggests that *O. lyallii* is alien to the Auckland Islands. Although such ‘native’ aliens can pose unique management challenges on islands, in this instance we suggest that ongoing monitoring with no control is an appropriate management action, as *O. lyallii* appears to pose minimal risk to ecological integrity.

## Abstract (precis)

Alien plants are a pervasive environmental problem, particularly on islands where they can rapidly transform unique indigenous ecosystems. However, often it is difficult to confidently determine if a species is native or alien, especially if establishment occurred before historical records. This can present a management challenge: for example, should such taxa be eradicated or left alone until their region of origin and status is clarified? In this paper we show how combining palaeoecological and historical records can help resolve such dilemmas, using the tree daisy *Olearia lyallii* on the remote New Zealand subantarctic Auckland Islands as a case study.

## Introduction

Alien plant species can pose a major threat to indigenous species, habitats and ecosystem function on islands, particularly if they become invasive (Vitousek 1988; Whittaker and Fernández-Palacios 2007). Although eradication or control is the common conservation response to invasive taxa (Simberloff *et al*. 2013), this requires a confident assessment that the targeted species is in fact alien. However, exactly what circumstances make a plant ‘alien’, let alone ‘invasive’ is unclear. Webb (1985) defines ‘native’ as a plant that has either evolved in a given place, or arrived at that place ‘entirely independently of human activity’. This definition is somewhat problematical, because a plant that can disperse long distances and arrive on an island without human assistance will be classified as a ‘native’, whereas another plant introduced by humans to the same place will be classified as ‘alien’. Both may be equivalent in terms of their ecological impact on that island. This can make it difficult to know how to manage the human-assisted movement and naturalization of plant species away from their natural biogeographic range, either between islands, or within larger islands, in an archipelago. For example, in New Zealand, there has been a tendency to regard any plant that is native within the archipelago to be native throughout, which defines the flora on the basis of political boundaries rather than biogeography. More recently, plants native to the New Zealand archipelago but growing out of their natural range are increasingly recognized as undesirable aliens that require control in conservation planning (e.g. Sawyer *et al*. 2003; DOC 2008).

Problems arise with regard to the practical application of the twin criteria (unaided movement and natural range) for native status in the face of climate change, and the biogeographic reality that many plants could have had a much larger ‘natural’ range before long-past events, such as the Last Glacial Maximum. Definitions that link alien status to anthropogenic dispersal can create conflicts for active management and global change mitigation strategies. The ‘Projected Dispersal Envelope’ concept of Webber and Scott (2012) argues for a definition that is based instead on the potential limit of a species, the distribution margin being determined by (i) the natural mechanisms that could move the dispersal unit the furthest distance within its native range and (ii) the time period regarded as relevant (e.g. post-glacial). Thus, if movement by natural mechanisms within the given time period is deemed impossible, then the organism is regarded as an alien within its new location. Another issue to consider is the ecological impact of the alien on its new range (Davis *et al*. 2011). In natural ecosystems, the problem with alien plant invasions is usually replacement, exclusion or suppression of native plants and detrimental changes to ecosystem function. However, where the alien is closely related to the species or even genotypes in the host area (as is often the case in interachipelago invasions), hybridization or genetic pollution is seen as the major threat (Godley 1972; Petit 2004).

In many cases, the region of origin for an alien species is clear, but often there is scope for confusion (Willis and Birks 2006). Palaeoecological records are increasingly recognized as a way to help determine a species’ region of origin and native/alien status with more confidence, by reconstructing the history of taxa over longer timescales than is possible through direct observations alone (Gillson *et al*. 2008). This approach works particularly well when fossil evidence for a species is morphologically unique, and species-specific baselines can be reconstructed with confidence (van Leeuwen *et al*. 2005). For example, pollen and macrofossil records have helped to resolve uncertainty around the native/alien status of numerous taxa on islands (e.g. van Leeuwen *et al*. 2005, 2008; Connor *et al*. 2012; Schofield *et al*. 2013).

**Figure 1.**
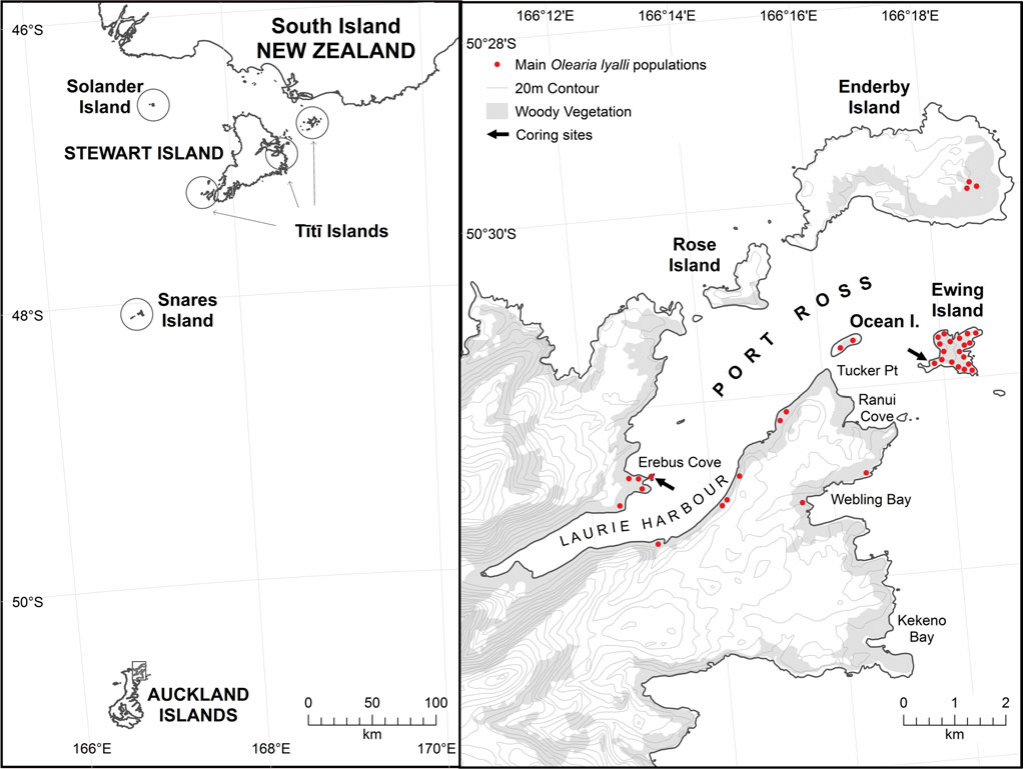
Map (left) showing the location of subantarctic Auckland Islands in relation to the South Island of New Zealand, and islands (circled) where *O. lyallii* currently occurs in New Zealand, and (right) the main *O. lyallii* populations on the north-eastern Auckland Islands (boxed area enlarged from map on left).

Well-dated, long-term and high-temporal resolution reconstructions of former vegetation composition can also show how, and under what ecological and environmental conditions, a species manages to invade and establish, and can determine the subsequent speed and spatial extent of spread (Gillson *et al*. 2008). The entire process of establishment and expansion can be documented through to the present, and then integrated with botanical or historical observations to develop a rich temporal and spatial perspective on an invasion, providing valuable insights for management practice and policy. We use this approach here to address the controversial status of a tree daisy *Olearia lyallii* (Asteraceae) on the Auckland Islands, a subantarctic island group in the New Zealand archipelago (Fig. 1). This tree is endemic to the New Zealand flora, but its origin and appropriate management on the Auckland Islands remains uncertain (Campbell and Rudge 1976; Lee *et al*. 1991; DOC 1998). By integrating palaeoecological records with historical evidence (written and photographic) and previous ecological investigations, we establish the history of *O. lyallii* arrival, establishment and subsequent spread on these islands. We also address the unresolved status of *O. lyallii* on the Auckland Islands according to the Projected Dispersal Envelope concept of Webber and Scott (2012) and determine whether its history and ecological role suggests that it poses a threat to the ecological integrity of the Auckland Island ecosystems.

### Background of *O. lyallii* on the Auckland Islands

The remote, uninhabited Auckland Islands (50.5°S) are afforded the highest level of protection status by the New Zealand Department of Conservation (DOC 1998). *Olearia lyallii* has a highly restricted distribution on small islands and adjacent coastal habitat in the northernmost Port Ross region of the Auckland Islands (Fig. 1). This includes all but the central parts of Ewing Island, where *O. lyallii* is thought to have initially established (Godley 1965), and at the short-lived Enderby Settlement site in Erebus Cove (Fig. 1) on the main Auckland Island, where canopy heights can reach 10 m. Elsewhere, there are isolated stands of a few trees or saplings scattered down the eastern side of Port Ross, Webling Bay and on Enderby and Ocean Island (Lee *et al*. 1991). A single tree has been recorded from Adams Island, the southernmost island of the Auckland Island archipelago, but has since been removed (Walls 2009). Lee *et al*. (1991), in their survey along the Laurie Harbour coastline, recorded ∼50 *O. lyallii* trees at nine sites. These scattered stands can be distinguished by their distinctive pale silvery foliage in Google Earth satellite imagery **[see Supporting Information—Figs S1 and S2]**.

**Figure 2.**
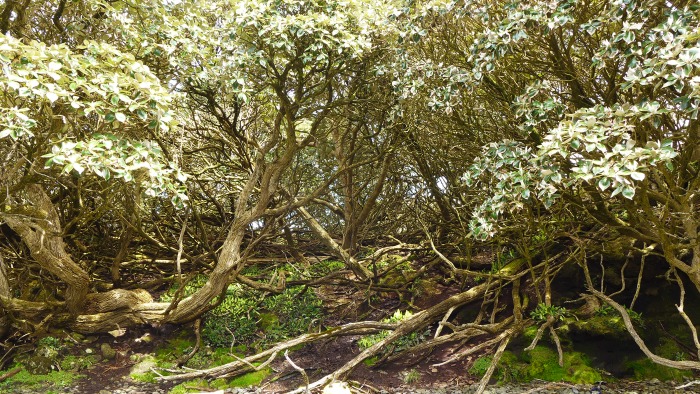
Photo (taken in 2013) of *O. lyallii* forest on the south west coast of Ewing Island, showing typically dense, tangled and shaded understory, with a ground cover of bare peat and ferns *Asplenium obtusatum* and *Blechnum durum*.

Elsewhere in the New Zealand region *O. lyallii* is confined to islands in a narrow 2° latitudinal belt, including the Snares Islands ∼270 km north of the Auckland Islands; coastal patches on Stewart Island; and on the Tītī and Solander Islands which are scattered around the coast of Stewart Island, ∼440 km to the north of the Auckland Islands (Fig. 1). Generally, *O. lyallii* dominated forests form dense, tangled and darkly shaded stands that suppress many lower-statured and light-demanding species leaving the sub-canopy almost devoid of any other plant species (Fig. 2). The tree has thick coriaceous leaves, is fast growing, nutrient demanding, flowers in profusion, is insect-pollinated and the seeds are wind-dispersed (Lee *et al*. 1991).

The status of *O. lyallii* on the Auckland Islands has been controversial since its discovery on Ewing Island off the main Auckland Islands in 1840 (Hooker 1844). It was first thought to be a remnant of a once more widely distributed forest (Hooker 1844; Cockayne 1909), but a more recent view is that it is an alien translocated in historic times from islands to the north (Godley 1965; Campbell and Rudge 1976). Campbell and Rudge (1976) saw *O. lyallii* as a threat to the native Auckland Island vegetation and argued for its control. In contrast, Lee *et al*. (1991) suggested that *O. lyallii* was causing minimal ecological impact and that regardless of how it arrived on the Auckland Islands, the tree was well within its natural dispersal limits and would have established on the islands eventually. On these grounds Lee *et al*. (1991) considered *O. lyallii* an acceptable biogeographic addition to the Auckland Island flora. The Department of Conservation's (DOC) management strategy (DOC 1998) for *O. lyallii* is cautious because of its unresolved native status. However, it is recognized that without any control, the current distribution of *O. lyallii* is likely to continue spreading slowly into, and reducing the area of, the maritime tussock–scrub–herbfield community that lies between the shore and the *Metrosideros* forest, particularly on the leeward side of the island (Lee *et al*. 1991). Department of Conservation's management strategy (DOC 1998) is for minimal control, but accompanied by monitoring of its distribution.

The Auckland Islands are well-suited to palaeoecological research. The islands are completely covered with thick (up to 12 m) organic peat deposits which have developed under a regime of high rainfall (2000 mm year^−1^) and a cool, cloudy and humid climate (McGlone 2002). Pollen and spores are well-preserved in the peats and allow detailed high-resolution vegetation reconstructions (McGlone *et al*. 2000; McGlone 2002), and current vegetation communities and species well-characterized by the modern pollen spectra (McGlone and Moar 1997).

The Auckland Islands are also well-documented from an historical point of view, with published observations of *O. lyallii* from 1840 onwards providing a chronological description of its habitat, stature and distribution (Hooker 1844; Cockayne 1909; Godley 1965; Campbell and Rudge 1976; Lee *et al*. 1991). The Auckland Islands were initially discovered by Polynesian voyagers in the 13th century, which is evidenced by earth ovens, shell and bone middens, stone flakes and scrapers and charcoal preserved in the sand dunes on Enderby Island (Fig. 1) (Anderson 2005). However, these early visitors did not settle on the islands permanently or leave any trace of their presence in the palaeo-vegetation or charcoal records (McGlone *et al*. 2000; Anderson 2005). Rediscovery of the islands by Europeans in 1806 marked the beginning of a short but intense period of disturbance and exploitation including: sealing and whaling from 1807; burning and small clearances, especially at Erebus Cove (Fig. 1) for the Enderby Settlement of 300 people between 1850 and 1852 and subsequent sheep grazing between 1874 and 1877 (Dingwall 2009); six major shipwrecks between 1833 and 1907 (Egerton *et al*. 2009); the introduction of alien mammals (pigs, cattle, rabbits, cats, mice, sheep, dogs and goats—of which only cats, pigs and mice now remain on the main Auckland Island); and the introduction of 37 plant species most of which are short-statured herbs and grasses of little threat to ecosystems or ecological processes (DOC 1998). The last ecological investigation of *O. lyallii* distribution and rate of spread was carried out in 1982, and predictions made of its likely trajectory (Lee *et al*. 1991).

The flora of the Auckland Islands is well-described (Cockayne 1909; Johnson and Campbell 1975). In the Port Ross region (Fig. 1), *Metrosideros umbellata* (Myrtaceae) forms a low forest with canopy heights ranging from 6 to 14 m where it is not exposed to strong winds or poor drainage **[see Supporting Information—Fig. S2]**. The small trees *Raukaua simplex*, *Myrsine divaricata*, *Dracophyllum longifolium* and *Coprosma foetidissima* are subdominant throughout the *Metrosideros* forest, reaching the canopy in tree-fall gaps, clearances or slips. They also occur on exposed coastal areas or in the upper forest-grassland ecotone. On coasts exposed to strong wind and salt spray, a maritime community of shrubland-grassland forms, including woody species: *Veronica elliptica*, *D. longifolium* and *Coprosma* spp.; graminoids: *Poa litorosa*, *P. foliosa* and *Carex appressa*; and ferns: *Polystichum vestitum* and *Asplenium obtusatum*. Where introduced pigs cannot get access, large-leaved forbs such as *Stilbocarpa polaris* and *Anistome latifolia* are prominent. *Metrosideros* forest is stunted or absent from the windward areas of Enderby Island and confined to the centre of Rose Island, Ocean Island and Ewing Island (Fig. 1).

## Methods

### Coring

We collected three peat cores from the Port Ross area, one from Ewing Island and two from Erebus Cove **[see Supporting Information—Figs S1 and S2]**. We used a hand-operated D-section corer to collect the Ewing Island core in 2013, which was taken from the southern end of the island to a maximum depth of sediment at 1.65 m (50°31′50.17″S; 166°17′50.43″E). The core site (Fig. 1) was under a mature monotypic coastal forest of *O. lyallii*, ∼2 m above sea level and ∼10 m from one of the few protected boat landings on the island.

**Figure 3.**
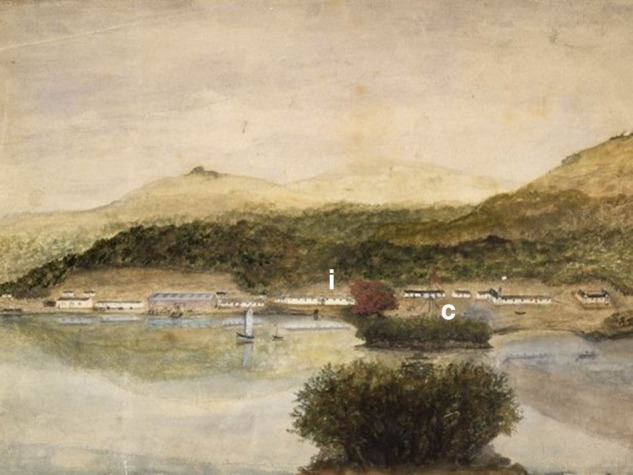
Painting by Charles Enderby, 1850–52, showing the clearing at Erebus Cove, the extent of the Enderby Settlement building, and the approximate locations of our coastal (c) and inland (i) core sites at Erebus Cove. The settlement flagstaff (later replaced with a signal mast) can be seen to the right of the red flowering *Metrosideros* tree and also marks the location of the coastal core site. The distinctive outcrop of Mt Eden can be seen on the hills to the south in the background. McNulty, Dorothy (Mrs), fl 1961. [Enderby, Charles] 1797–1876. Attributed works. :[Port Ross, Auckland Islands, Between 1850 and 1852?]. Ref: A-093-008. Alexander Turnbull Library, Wellington, New Zealand. http://natlib.govt.nz/records/23243247.

**Figure 4.**
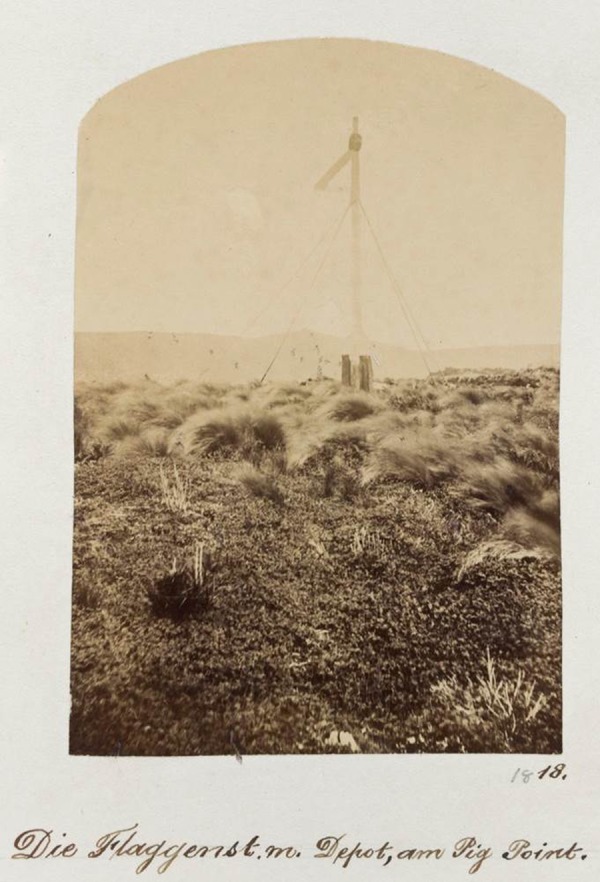
Historic photograph of Erebus Cove taken by G. Wolfram in 1874, showing the former site (abandoned and dismantled) of the Enderby Settlement; the signal mast (the same one shown in Fig. 3) that replaced the Enderby Settlement flagstaff; and the tussock grasses which were grazed by Monckton's sheep between 1874 and 1877. An abundant patch of herbaceous *Acaena* can be seen in the foreground, and the Mt Eden outcrop on the hills in the background (between the supporting wires on the left). Photo one of many taken by G. Wolfram in 1874, courtesy of State Library of Victoria, Melbourne (H86.2/9).

At Erebus Cove, two short peat cores were collected in 2008 by digging pits and pushing a half-drainpipe into the wall of the peat sections. One of these Erebus Cove cores, labelled ‘coastal Erebus Cove’ (51 cm deep), was collected ∼5 m from the shoreline under a forest co-dominated by mature *O. lyallii* and *M. umbellata* trees ∼2 m above sea level (50°32′45.76″S; 166°12′55.22″E). This coring site is adjacent to the Enderby Settlement flagstaff (later replaced with a signal mast), which can be seen in many early historic photos and paintings of Erebus Cove (e.g. Figs 3 and 4).

The second Erebus Cove core, labelled ‘inland Erebus Cove’ (53 cm deep), was taken from under *M. umbellata* forest canopy with a *Dracophyllum*, *R. simplex* and *Coprosma* understory. The site was ∼500 m inland from the coastal *O. lyallii* site (50°32′48.94″S; 166°12′46.09″E). The approximate locations of both Erebus Cove cores are marked on a historic painting from 1850 of the Enderby Settlement (Fig. 3).

All cores comprised coarse, fibrous, poorly humified, highly organic, red-brown peat. The cores were wrapped in the field, and sub-sampled in a clean laboratory environment. We sampled for pollen and charcoal to a depth of 100 cm in the Ewing Island core, and to the base of the Erebus Cove cores.

### Microscopic pollen and charcoal analyses

We used standard treatments of highly organic peats (KOH, acetolysis, and filtering through a 100 µm mesh sieve) to prepare microscopic pollen slides (Moore *et al*. 1991). We counted pollen and spores on each slide until we had recorded at least 250 grains from terrestrial plants (the pollen sum) from which percentages were calculated. We have used the recommended nomenclature for New Zealand pollen taxonomic groups (Moar *et al*. 2011). Statistical differences in composition between pollen zones were estimated with a non-parametric permutational multivariate analysis of variance (PERMANOVA) (Anderson 2001) using adonis in the vegan library with default settings, Bray's distance measure, and 9999 permutations. We log-transformed the pollen data while preserving zero values following (McCune *et al*. 2002). Results were compared with those using analyses of multivariate abundance using the function many.lm in the R library mvabund (Wang *et al*. 2012).

For the Ewing Island core we reconstructed local fire history following standard charcoal-analysis procedures (Whitlock and Larsen 2001), counting all charcoal particles present in a 1 mL sample that were retained on nested sieves of 125 and 250-µm mesh size. For the Erebus Cove cores, we counted microscopic charcoal particles on the pollen slides (Clark 1982), which we expressed as a percentage of the total number of pollen grains counted. Although we have used two different techniques to record charcoal presence in the Ewing Island and Erebus Cove cores, our work from subantarctic Campbell Island has shown that microscopic and macroscopic charcoal records are highly correlated (McGlone *et al*. 2007).

### Pollen identification

Pollen analysis of surface samples taken under different vegetation types on the Auckland Island show that broad communities can be distinguished by characteristic pollen taxa (McGlone and Moar 1997). *Olearia lyallii* is closely related to three Asteraceae herb species in the *Pleurophyllum* genera found on the Auckland Islands. While *O. lyallii* pollen can easily be distinguished from *P. speciosum*, it cannot always be reliably distinguished from *P. hookeri* type (including *P. hookeri* and *P. criniferum*) (Moar *et al*. 2011) **[see Supporting Information—Fig. S3]**. However, on the Auckland Islands *P. hookeri* is rarely found at sea level, is only common above 450 m in mountain tundra communities where is rarely exceeds 5 % of the pollen sum; and *P. criniferum*, although found in maritime communities, rarely makes up >0.5 % of the pollen sum. We use these modern abundances as a guide to provide a level of confidence on the *O. lyallii* pollen curves, placing a 5 % reference baseline on our pollen diagrams. None of the *Pleurophyllum* species occurs under a forest canopy, and the palatable *P. criniferum* is scarce in the presence of pigs, which are common at Erebus Cove. In contrast to *Pleurophyllum* pollen representation, surface samples taken from under an *O. lyallii-*dominant forest on the Snares Island and Ewing Island show that *O. lyallii* pollen makes up 50 and 80 % of the pollen sum, respectively **[see Supporting Information—Table S1]**. The greater number of flowers and therefore pollen produced by *O. lyallii* compared with herbaceous *Pleurophylum* spp. per unit area sampled results in significantly higher pollen percentages under an *O. lyallii* canopy.

### Radiocarbon dating

Peat samples were taken from the cores (1 cm vertical thickness) and submitted for Accelerator Mass Spectrometry (AMS) radiocarbon dating at the Waikato Radiocarbon and Beta Analytic Dating Laboratories (Table 1), with eight dates from the Ewing Island core, and two from each of the Erebus Cove cores. Radiocarbon ages were calibrated using OxCal (Ramsey 2008) using the SHCal13 calibration dataset (Hogg *et al*. 2013). Modern radiocarbon ages (i.e. post 1950 AD) were calibrated using Calibomb (http://calib.qub.ac.uk/CALIBomb (accessed 2015); using SHCAL 13 and SHZ1-2 bomb extension zone options). We calculated an age-depth model for the Ewing Island core using the P_sequence option in OxCal **[see Supporting Information—Table S2 and Fig. S4]**. Using Bayes theorem, the algorithms employed possible solutions with a probability that is the product of the prior and likelihood probabilities. The posterior probability densities quantify the most likely age distributions. The OxCal outlier model (*A*_model_= 98.9; *A*_overall_= 99.5) identified one date (BETA-395476) as an outlier that was removed from the model. All calibrated ages are reported here as calendar (cal) years AD (Table 1). We estimated the time for the first appearance of *O. lyallii* pollen in the coastal Erebus Cove core using linear interpolations between the two calibrated dates from this core **[see Supporting Information—Table S3]**.

**Table 1.** Radiocarbon dates from Ewing Island and Erebus Cove peat cores, Auckland Islands. Calibrations based on Southern Hemisphere Calibration Curve (SHCAL13) from Hogg *et al*. (2013). **Identified as an outlier in age-depth model **[see Supporting Information—Fig. S4]** and *modern dates on Calibomb (http://calib.qub.ac.uk/CALIBomb).

Core site (and laboratory code)	^14^C Lab code	Depth (cm)	Conventional C^14^ age	Dated material	AD calibrated years 1 sigma calibration (with relative area) and most likeliest age with probability highlighted
Ewing Island south, Coastal *Olearia* (site X13/84)	BETA-395475	10	116.5 ± 0.3	Peat	1959 (0.06)
					1960 (0.02)
					1963 (0.002)
					1988 (0.08)
					**1989–91 (0.7)**
					1991 (0.09)
					1992 (0.04)
	BETA-400420	25	130 ± 30	Plant remains	1705–21 (0.13)
					1810–37 (0.24)
					1845–66 (0.16)
					**1879–1931 (0.46)**
					1939–42 (0.01)
	**BETA-395476	33	670 ± 30	Peat	Not calibrated (age inversion)
	BETA-400421	45	290 ± 30	Plant remains	1518–38 (0.16)
					**1626–68 (0.81)**
					1788–92 (0.03)
	BETA-395477	75	750 ± 30	Peat	**1274–1302 (0.82)**
					1365–75 (0.18)
	BETA-400422	80	720 ± 30	Plant remains	**1286–1312 (0.56)**
					1359–80 (0.45)
	BETA-400423	148	3790 ± 30	Peat	**BC 2205–2129 (0.73)**
					2087–48 (0.27)
	Wk-38432	165	8768 ± 27	Peat	**BC 7789–7648 (1)**
Auckland Is, Inland Erebus Cove *Metrosideros* (site X08/22)	*Wk-31424	5	100.3 ± 0.4	Peat	1955 (0.29)
					**1955–56 (0.70)**
					1956 (0.009)
	Wk-31425	50	275 ± 27	Peat	**1635–70 (0.84)**
					1749–52 (0.03)
					1784–94 (0.13)
Auckland Is, Coastal Erebus Cove, *Olearia* (site X08/23)	*Wk-31426	5	116.50 ± 0.4	Peat	1958–59 (0.1)
					1995–95 (0.2)
					**1996–98 (0.7)**
	Wk-31427	50	31 ± 27	Peat	1890–1910 (0.36)
					**1815–30 (0.33)**

For the historical ecology, we examined published accounts of various botanical excursions to the islands (including: Hooker 1844; Chapman 1891; Cockayne 1903, 1905, 1909; Godley 1965; Campbell and Rudge 1976; Smith 2002) and examined photos and paintings of the Port Ross area of Auckland Island from electronic archives, including the Museum of New Zealand Te Papa Tongarewa, Alexander Turnbull Library (New Zealand) and State Library of Victoria (Australia).

## Results

For ease of interpretation, we have divided the Ewing Island and coastal Erebus Cove profiles into two zones: the uninvaded zone and *Olearia* zone, the latter defined by the first presence of *O. lyallii* pollen. The inland Erebus cove pollen profile is divided into the pre- and post-Enderby Settlement zones, the latter defined by the decline and subsequent regeneration of *Metrosideros* forest.

### Ewing Island core

**Figure 5.**
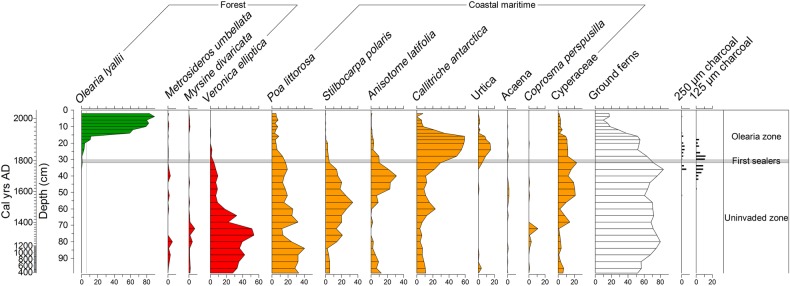
Summary percentage pollen record from Ewing Island, with pollen taxa plotted against depth, with the calibrated age scale in years AD shown on the secondary axis. Grey zone shows time of earliest sealing activity in the region (1807–10).

The radiocarbon dates (Table 1) and age-depth model for this core **[see Supporting Information—Table S2 and Fig. S4]** indicate peat accumulation began on Ewing Island c. 10 000 cal year before the present. The base of our pollen record (Fig. 5) starts at c. 1600 cal year before the present (c. 400 cal year AD), at which time the site was covered with a coastal maritime community dominated by the shrub *V. elliptica*, with grasses, macrophyllous forbs *S. polaris* and *A. latifolia*, sedges, and abundant ground ferns. The low levels of *Metrosideros* pollen throughout the core suggests that this forest was limited to the more protected interior of the island behind the coastal belt of maritime vegetation. Low counts of charcoal (<5 fragments per 1 mL of peat) are first recorded in the peat profile at the top of the uninvaded zone, just prior to c. 1800 cal year AD but probably reflect reworking as a consequence of site disturbance during the European era (also supported by age inversion at 33 cm—Table 1).

At the base of the *Olearia* zone at 32 cm, further charcoal particles, and the first trace of *O. lyallii* occur at an estimated age of c. 1800 cal year AD **[see Supporting Information—Table S2]**. At the same time, there is an increase of the herbs *Callitriche antarctica* and *Urtica australis*, and a decline of grasses, *S. polaris* and *A. latifolia*. Pollen of *O. lyallii* remains below 5 % of the pollen sum until 1870 cal year AD after which time it increases towards the top of the core, while ground ferns, herbs and grasses decline. As the *O. lyallii* canopy matured and closed creating a dense shade, it suppressed many lower-statured and light-demanding plants, leaving the sub-canopy and canopy floor almost bare. The pollen composition in the uninvaded and *Olearia* zone was significantly different (*F*_1,28_ = 27.4, *P* < 0.001). Similar, statistically strong differences in composition were detected using an analysis of multivariate abundance using the function many.lm in the R library mvabund (Wald statistic = 26.15, *P* = 0.001).

### Erebus Cove cores

**Figure 6.**
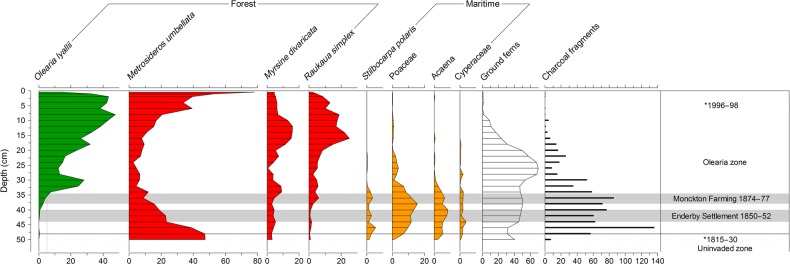
Summary percentage pollen record from the coastal Erebus Cove site (taken under an *O. lyallii* canopy), main Auckland Island. Grey bars show the time of the Enderby Settlement (1850–52) and Monckton Farming (1874–77) periods according to age-depth model. *Position and age of calibrated radiocarbon (cal year AD) dates from Table 1.

**Figure 7.**
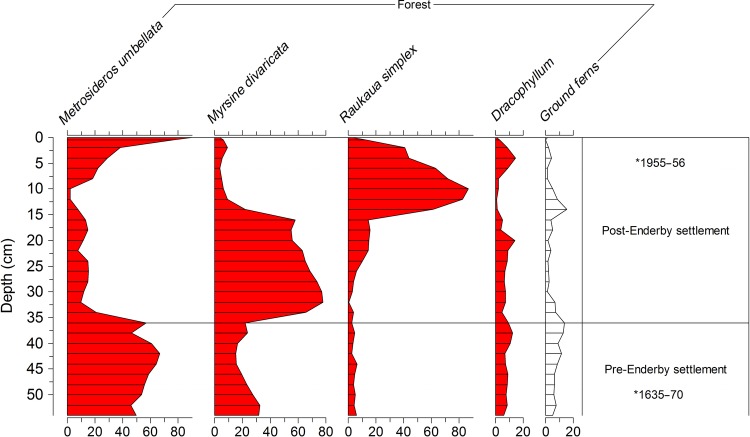
Summary percentage pollen record from the inland Erebus Cove site (taken under a *M. umbellata* canopy 500 m inland from the coastal Erebus Cove site) main Auckland Island. *Position and age of calibrated radiocarbon dates (cal year AD) from Table 1.

The uninvaded and pre-Enderby Settlement zones of both the coastal and inland Erebus Cove cores, respectively, record a *Metrosideros-*dominated forest (constituting ∼40–50 % of the pollen sum). The coastal site (Fig. 6) has a greater representation of ground ferns, *S. polaris*, *Acaena* and grass, reflecting its more open canopy. In contrast, the inland site (Fig. 7) has a substantial representation of the small trees *R. simplex*, *D. longifolium* and *M. divaricata*.

Both the coastal and inland Erebus Cove pollen profiles show a rapid and marked reduction of *Metrosideros* at 45 and 36 cm, respectively, accompanied by an abundance of charcoal fragments at the coastal site. This forest decline reflects the burning and cutting of trees at the coastal site, and felling of trees at the inland site to make way for the Enderby Settlement in 1850, as shown in paintings and photos from this period (e.g. Figs 3 and 4).

The calibrated date from the base of the coastal Erebus Cove core provides two age ranges (1910–1890 and 1830–15 cal year AD) with equal probability distributions (Table 1). As botanists did not record *O. lyallii* trees at Erebus Cove during a visit in 1890 (Chapman 1891), we take the older of the two solutions to create an age-depth model (using linear interpolation between this date and the 1955–56 cal year AD date at 5 cm **[see Supporting Information—Table S3]**). This model provides an estimate for the first trace of *O. lyallii* pollen (0.5 % at 48 cm) of 1823–37 cal year AD. *Olearia lyallii* pollen is not recorded again until 42 cm (c. 1847–60 cal year AD) after the *Metrosideros* has been burnt and cleared. After this, *O. lyallii* is consistently recorded, increasing to a peak of 47 % (Fig. 6) and later declining as *Metrosideros* begins to recover at the site.

At the inland Erebus Cove site (Fig. 7) charcoal and *O. lyallii* pollen are not recorded following the decline of *Metrosideros*. Instead, after forest clearance, a woody succession takes place from *M. divaricata* through *R. simplex* to *Metrosideros*, the latter recovering mainly through sprouting from cut stumps which are still visible in the forest today.

Pollen composition was significantly different in the pre- and post-settlement zones of the inland Erebus Cove core (*F*_1,27_ = 7.33, *P* < 0.0001). Similar, statistically strong differences in composition were detected using an analysis of multivariate abundance using the function many.lm in the R library mvabund (Wald statistic = 116.9, *P* < 0.0001). However, as there was only one sample from the uninvaded period from the coastal Erebus Cove core, statistical comparisons of compositional variance in the uninvaded and *Olearia* zones are very weak and non-significant (PERMANOVA: *F*_1,25_ = 1.47, *P* = 0.18 and multivariate generalized linear models Wald statistic = 7.38, *P* = 0.22).

### Earliest historical documentation of *O. lyallii* on the Auckland Islands

Written and photographic records from the 19th century to the mid-20th century provide a surprisingly detailed account of *O. lyallii* on the Auckland Islands (e.g. Hooker 1844; Chapman 1891; Cockayne 1903, 1905, 1909; Godley 1965; Campbell and Rudge 1976). The first record of the tree was the type specimen collected from Ewing Island by David Lyall in 1840, a botanist on the Ross Expedition. Hooker (1844:38) described the specimen as follows: ‘…a short stout trunk rises a few inches above the ground, and then sends off horizontally patent branches, which radiate as from a common centre for 10 to 12 feet on all sides, a little above the surface of the earth.’ Hooker (1844) further describes *O. lyallii* as rare on the Auckland Islands, and McCormick (1884), who also landed on the island at the same time as Lyall, did not mention seeing this species. Godley (1965) remarked that these descriptions typically matched stunted plants in exposed locations, but a canopy of at least 6 m in diameter would suggest the specimen described by Hooker (1844) had been growing for some years. However, as the *O. lyallii* specimen was not flowering (despite being collected in early summer) and low-growing, Campbell and Rudge (1976) suggested on the basis of observations of *O. lyallii* growing on the Snares that this tree may have been <20 years old when first seen by Lyall in 1840.

## Discussion

### Timing, dispersal and origin of *O. lyallii* on Auckland Islands

The estimated ages for the first appearance of *O. lyallii* pollen in our dated pollen profiles indicate that this tree daisy established on Ewing Island ∼1800, and then later at Erebus Cove c. 1823–37, exceeding >5 % of the pollen sum at both sites <60 years later. Despite the limited precision expected of radiocarbon dates from fibrous peat, this timing is consistent with historical observations (Godley 1965; Smith 2002; Prickett 2009) that strongly suggest initial establishment on Ewing Island c. 1807–10. Sealers were active at this time within the natural range of *O. lyallii* on the Snares Island and the 36 smaller Tītī islands around Stewart Island, as well as on the northern Auckland Islands (Smith 2002). Sealing activities caused localized disturbance to the mainly coastal flora and fauna (Smith 2002), and undoubtedly increased the possibilities for seed translocation between islands. Our age estimates are also consistent with age–diameter relationships made in 1982 of one of the largest erect *O. lyallii* trees on Ewing Island located close to an old whaling boat shed. This sampled tree had a trunk diameter of 110 cm indicating establishment ∼1820 (Lee *et al*. 1991).

It has been proposed that *O. lyallii* had the capacity to eventually disperse to the Auckland Islands through natural agencies and fill a previously vacant niche (Lee *et al*. 1991). *Olearia lyallii* seeds are adapted for transport by wind and can also potentially attach to feathers of ground nesting sea birds; the 4-mm long achenes have 6-mm long fluffy pappus hairs (Allan 1982) and are produced in abundance. The Auckland Islands are also only 270 km from the source islands of *O. lyallii* which also harbour large populations of nesting sea birds. Thus, this tree daisy has had numerous opportunities during the Holocene to disperse naturally to the Auckland Islands. However, our pollen records, and other pollen records from the islands (McGlone *et al*. 2000; McGlone 2002), show that *Metrosideros* forest, and coastal maritime communities have dominated sheltered and exposed coastal habitats, respectively, on the Auckland Islands for at least 12 000 years. *Olearia lyallii* has only managed to establish in a few scattered places in the northern Auckland Islands in the last 200 years, coincident with the earliest European exploitation of the region. We conclude from this evidence that there is a high probability that the Auckland Islands lie outside of the natural distribution range of *O. lyallii*, and require human-assisted seed dispersal and/or new niches in order to establish.

### Process of initial *O. lyallii* establishment on Ewing Island

The pollen and charcoal record from Ewing Island (Fig. 5) shows that the initial establishment of *O. lyallii* on this island was into an anthropogenically disturbed habitat, not into a pristine coastal maritime community. Fire-induced changes to the coastal vegetation preceded the establishment of *O. lyallii*, suggesting facilitation by anthropogenic disturbance. Charcoal is almost absent from all subantarctic island Holocene peat records until the arrival of Europeans (McGlone *et al*. 2000; McGlone 2002; Wilmshurst *et al*. 2004; Bestic *et al*. 2005).

Sealers often used overland routes to access sealing spots as the seas were rough and dangerous, and New Zealand fur seals (*Arctocephalus forsteri*) and Hooker's sea lions (*Phocarctos hookeri*) dispersed along the coast (Smith 2002). Walking through the dense and tangled vegetation of the Auckland Islands is notoriously slow and arduous, and fire was used liberally by 19th century travellers to clear the way for easier travel. For instance, officers from the 1840 Terror and Erebus expedition to the islands set fire to forest and scrub in the hills immediately above Erebus Cove. Robert McCormick from the same expedition, in his excursion from Terror Cove to Matheson Bay and around the peninsula to Deas Head, noted that there was extensive burnt grassland on the cliffs (McCormick 1884). The earliest charcoal presence in the Ewing Island core precedes the earliest shipwrecks in the Auckland Islands (from 1888 to 1907) (Egerton *et al*. 2009) and suggests that the Ewing Island coring area had been burnt by sealing gangs to ease their passage through dense coastal vegetation to reach seal haul-out sites. The pollen and charcoal record from Ewing Island also shows that during the first few decades of *O. lyallii* establishment, the herbs *U. australis* and *C. antarctica* became abundant, indicating a succession similar to that recorded on the Snares Island following dieback of *Veronica* and subsequent abandonment of penguin colonies (Hay *et al*. 2004). *Callitriche antarctica* commonly colonizes abandoned penguin colonies on the Snares Islands (Hay *et al*. 2004), and *U. australis* responds favourably to disturbance and high-light (Allan 1982). These nutrient-demanding herbs likely established on the disturbed and marine-enriched patches of bare peat on Ewing Island that were previously maintained by seals in haul-out areas along the coast. As vast numbers of seal carcasses were usually left to rot *in situ* where the animals were slaughtered on these coastal habitats (Smith 2002) their decomposing bodies may have provided a pulse of nutrient enrichment during the time of *O. lyallii* establishment. Outside of current *O. lyallii* forest patches on the Auckland Islands, *O. lyallii* seedlings are most commonly recorded on recently abandoned sea lion haul-outs with bare patches of peat (Lee *et al*. 1991).

The presence of ground nesting and burrowing sea birds on Ewing Island may also explain why *O. lyallii* has been so successful on Ewing Island compared with elsewhere on the Auckland Islands, as they also provide substantial and continuing sources of disturbance and nutrients to the Ewing Island peats. On the main Auckland Islands and other smaller islands in the Port Ross area, introduced pigs, cattle, cats (Challies 1975; Lee *et al*. 1991) and potentially mice (c.f. Cuthbert and Hilton 2004), have almost completely eliminated nesting sea bird populations (through trampling and predation), and therefore associated marine nutrient transfer. In contrast, Ewing Island has remained free of introduced mammals. The continued input of marine-derived nutrients from sea birds during the early human disturbance phase, and later recovery of seal populations, has likely promoted *O. lyallii* establishment on Ewing Island, and fuelled its rapid growth rates.

On Ewing Island, as the *O. lyallii* canopy became taller and more open as it matured, and bulky leaf litter built up thick peat deposits, these conditions would have become increasingly attractive for nesting sea birds where they could burrow and land/take-off easily (see Whitehead *et al*. 2014). The input of marine nutrients by seals and sea birds, and the dark shade and rapid growth of *O. lyallii* forests on enriched soils may allow this tree to exclude the former coastal maritime communities indefinitely. *Olearia lyallii* benefits from marine-enriched soils in its natural range on the Snares, Solander and Tītī islands (Fig. 1). These islands are wind-swept, drenched with salt spray during storms and largely covered with organic peat deposits which are extensively burrowed and disturbed by nesting sea birds and seal activity. The strong fertilizing effect of marine animals on the soils is reflected in the *O. lyallii* leaves from the Snares Islands which have yielded some of the highest leaf ^15^N enrichment levels ever recorded for plants (Martinelli *et al*. 1999; Hawke and Newman 2007). However, away from the smaller islands, on nearby Stewart Island nesting sea bird densities are much lower and *O. lyallii* has a very limited distribution (Wilson 1987). *Olearia lyallii* distribution is almost certainly dependent on marine subsidies introduced by sea birds and seals.

### Rates of *O. lyallii* spread on Ewing Island

Historical observations and the pollen record from Ewing Island suggests that it took *O. lyallii* ∼80 years to shade out the tall tussock-and herbaceous maritime communities (Fig. 5). In 1840, 20 years after its establishment, *O. lyallii* was described as quite rare and stunted among the maritime tussock and scrub on Ewing Island (Hooker 1844). By 1890, substantial trees of *O. lyallii* were present along the sea shore, but large tussocks remained common (Chapman 1891). In 1907, a low *O. lyallii* forest appears to have extended over most of the island aside from the central *Metrosideros* core, but patches of tall tussocks apparently were still present (Godley 1965). By the 1960s the tree daisy had formed a coastal fringe around the island (Godley 1965). The slow spread of *O. lyallii* into the *Metrosideros* forest over the last 50 years suggests a superior competitiveness of *O. lyallii* on nutrient-enriched soils in exposed locations. Although *Metrosideros* is a long-lived tree, and can resprout or layer after damage, it is shade-intolerant and slow growing (Wardle 1971) and vulnerable to over-topping by *O. lyallii* where wind and salt exposure causes shorter-statured canopies.

### Timing of *O. lyallii* establishment at Erebus Cove

Historical observations also suggest *O. lyallii* has made a slow and limited spread away from Ewing Island to other sites on the main Auckland Island and smaller islands in the Port Ross area (Campbell and Rudge 1976; Lee *et al*. 1991). The age estimate for the first trace of *O. lyallii* pollen in the dated coastal Erebus Cove pollen profile is 1823–37 cal year AD (Fig. 6), some 15–40 years after it is recorded at our site on Ewing Island. These first traces of *O. lyallii* may reflect pollen contributed from scattered individuals in the coastal communities at Erebus Cove that did not initially succeed, or possibly *P. criniferum*, which can occur in coastal communities at trace levels (McGlone and Moar 1997). The pollen type is not recorded again until 42 cm in the profile (c. 1847–60) after which it steadily increases to a peak of 47 %. However, the precision of these age estimates is limited, as they are only based on linear interpolation between two dates, the lowest of which has a spread of calibrated age ranges spanning 75 years, therefore we use the historical evidence to refine the timing of *O. lyallii* establishment at Erebus Cove.

The conjecture has been that colonists at the Enderby Settlement transplanted *O. lyallii* from Ewing Island to this site, either accidentally or as an ornamental, at some time between 1850 and 1852 (Godley 1965; Campbell and Rudge 1976). The extent of clearance for the Enderby Settlement (1850–52) can be seen in the painting of Erebus Cove in c. 1850 by Charles Enderby (Fig. 3). In 1865, Captain Musgrave visited the Enderby Settlement site at Erebus Cove and made reference to ‘two trees’ (Musgrave 1866) which have been interpreted by Campbell and Rudge (1976) as being *O. lyallii* specimens. However, shortly after the Enderby Settlement dismantled, there was a brief farming episode (September 1874–May 1877) by Monckton, a lease-holder at Erebus Cove (Dingwall 2009), when 56 sheep were grazed on the cleared site, scrub and grass was burnt, and grass and oats were planted (Dingwall 2009). A photograph in 1874 of the signal mast that replaced the original settlement flagstaff on Davis Point (the coastal Erebus core site) shows a landscape dominated by tall grass, with a mat of the herb *Acaena* in the foreground, but with no sign of any trees (Fig. 4). This corresponds well with the *Acaena* pollen recorded from this site, after the forest clearance but before the *O. lyallii* invasion (Fig. 6). Chapman and Kirk visited Erebus Cove in 1890, and they did not record *O. lyallii* (Chapman 1891). Only in 1907, some 55 years after the Enderby Settlement, and 30 years after the Monckton lease, there is evidence for *O. lyallii* at Davis Point near the flagstaff (Godley 1965). Cockayne (1909) mentions ‘a few trees in the neighbourhood of the Port Ross depot’. If *O. lyallii* had been introduced by the Enderby Settlement colonists to Erebus Cove, these trees had made little growth. From these observations it seems more likely that *O. lyallii* established during or after the Monckton farming interval (1874–77).

### Process of establishment at Erebus Cove

The pollen record from the coastal Erebus Cove site (Fig. 6) is similar in one key respect to the Ewing Island record, in that *O. lyallii* did not establish into a pristine *Metrosideros* forest but into a burned and disturbed coastal maritime community dominated by grasses and the herbs *Acaena* and *S. polaris*, and with low levels of the successional small trees *M. divaricata* and *R. simplex*. However, it differs significantly from Ewing Island, in that as the *O. lyallii* canopy matured towards the present, it became co-dominant with the recovering *Metrosideros*. As there are almost no nesting sea birds at Erebus Cove and limited seal presence, *O. lyallii* lacks its preferred enriched peat. This sheltered site favours *Metrosideros* which is now co-dominant with *O. lyallii* and, being longer-lived, will eventually over-top and replace it.

There is no evidence from the pollen record for *O. lyallii* presence at the inland Erebus Cove site following abandonment of the Enderby Settlement (Fig. 7), despite being only <500 m away from the well-established population at the coastal site. Instead, the pollen record shows a succession from *Myrsine* to *Raukaua* and back to the pre-settlement *Metrosideros* forest. Seed dispersal by wind, people or animals over such a short distance cannot have limited *O. lyallii* establishment at the inland site or indeed elsewhere in the Port Ross area. However, nutrients derived from marine aerosols drop off rapidly with distance from the shore (Meurk *et al*. 1994), and without marine nutrient subsidies and disturbance, *O. lyallii* loses its competitive advantage.

## Conclusions

Despite having ample opportunity to disperse to the Auckland Islands from the small island groups to the north (Snares, Solander, Tītī and Stewart Islands), a combination of palaeoecological and historical observations suggests that *O. lyallii* is only a recent addition to the flora of the Auckland Islands. It was most likely introduced by sealers between 1807 and 1810. Under the Projected Dispersal Envelope concept of Webber and Scott (2012) regarding natural dispersal and time, it is unambiguously an alien plant on the Auckland Islands. Despite its alien status, our observations indicate that *O. lyallii* is not highly invasive, and poses little threat to the ecological integrity of the island, in agreement with Lee *et al*. (1991). Detailed palaeoecological records have shown that the establishment of *O. lyallii* on the Auckland Islands was facilitated by human disturbance; that its spread has been slow; and its distribution limited to exposed coastal habitats where peats have been enriched by sea birds, seals and salt spray. Given the limited distribution of anthropogenically disturbed and enriched habitats on the islands, *O. lyallii* is unlikely to pose a significant threat to the existing maritime habitat on the uninhabited islands, and no threat to the *Metrosideros* forest.

Climate change and the inevitable human-assisted movement of propagules across landscapes will ensure that the issue of ‘native’ aliens will arise repeatedly. However, palaeoecological and historical research such as presented here and elsewhere (e.g. van Leeuwen *et al*. 2008) can help conservation agencies make considered decisions regarding the management and status of such plants (Gillson *et al*. 2008). We support the dynamic and pragmatic ‘monitor and see’ approach for *O. lyallii* (Davis *et al*. 2011) that balances what appears to be a limited loss of ecological integrity with the high cost and low probability of successful control.

## Sources of Funding

Our study was supported by Core Funding for Crown Research Institutes, from the New Zealand Ministry of Business, Innovation and Employment's Science and Innovation Group.

## Contributions by the Authors

J.M.W. and M.S.M. conceived the study, collected cores, analysed the cores and wrote the paper. C.S.M.T. contributed to the writing of the paper and calculated the age-depth models.

## Conflict of Interest Statement

None declared.

## Supporting Information

The following additional information is available in the online version of this article –

**Figures S1 and S2.** Location and vegetation cover of Ewing Island and Erebus Cove coring sites.

**Figure S3.** Microphotographs of *Olearia lyallii* and *Pleurophyllum* spp. pollen grains.

**Figure S4.** Output graph for Ewing Island age-depth model.

**Table S1.** Modern pollen percentages from surface samples under *Olearia lyalli-*dominated canopies.

**Table S2.** Age-depth model for Ewing Island core.

**Table S3.** Linear interpolation between calibrated radiocarbon ages in coastal Erebus Cove core.

Additional Information

## References

[PLV104C1] Allan HH 1982 Flora of New Zealand Volume 1. Wellington: P.D. Hasselberg, Government Printer.

[PLV104C2] Anderson A 2005 Subpolar settlement in South Polynesia. Antiquity 79:791–800.

[PLV104C3] Anderson MJ 2001 A new method for non-parametric multivariate analysis of variance. Austral Ecology 26:32–46.

[PLV104C4] Bestic KL, Duncan RP, McGlone MS, Wilmshurst JM, Meurk CD 2005 Population age structure and recent *Dracophyllum* spread on subantarctic Campbell Island. New Zealand Journal of Ecology 29:291–297.

[PLV104C5] Campbell DI, Rudge MR 1976 A case for controlling the distribution of the tree daisy (*Olearia lyallii*) Hook.F. in its type locality, Auckland Islands. Proceedings of the New Zealand Ecological Society 23:109–115.

[PLV104C6] Challies CN 1975 Feral pigs (*Sus scrofa*) on Auckland Island: status, and effects on vegetation and nesting sea birds. New Zealand Journal of Zoology 2:479–490. 10.1080/03014223.1975.9517889

[PLV104C7] Chapman FR 1891 The outlying islands of New Zealand. Transactions of the New Zealand Institute 23:491–522.

[PLV104C8] Clark RL 1982 Point count estimation of charcoal in pollen preparations and thin sections. Pollen et Spores 24:523–535.

[PLV104C9] Cockayne L 1903 Botanical excursion during midwinter to the southern islands of New Zealand. New Zealand Institute.

[PLV104C10] Cockayne L 1905 Notes on a brief botanical visit to the Poor Knights Islands. Transactions and Proceedings of the New Zealand Institute 38:351–360.

[PLV104C11] Cockayne L 1909 The ecological botany of the subantarctic islands of New Zealand. In: Chilton C, ed. The Subantarctic Islands of New Zealand. Christchurch: Philosophical Institute of Canterbury.

[PLV104C12] Connor SE, van Leeuwen JFN, Rittenour TM, van der Knaap WO, Ammann B, Björck S 2012 The ecological impact of oceanic island colonization–a palaeoecological perspective from the Azores. Journal of Biogeography 39:1007–1023. 10.1111/j.1365-2699.2011.02671.x

[PLV104C13] Cuthbert R, Hilton G 2004 Introduced house mice *Mus musculus*: a significant predator of threatened and endemic birds on Gough Island, South Atlantic Ocean? Biological Conservation 117:483–489. 10.1016/j.biocon.2003.08.007

[PLV104C14] Davis MA, Chew MK, Hobbs RJ, Lugo AE, Ewel JJ, Vermeij GJ, Brown JH, Rosenzweig ML, Gardener MR, Carroll SP, Thompson K, Pickett STA, Stromberg JC, Tredici PD, Suding KN, Ehrenfeld JG, Grime JP, Mascaro J, Briggs JC 2011 Don't judge species on their origins. Nature 474:153–154. 10.1038/474153a 21654782

[PLV104C15] Dingwall PR 2009 Pastoral farming at the Auckland Islands. In: Dingwall PR, Jones KL, Egerton R, eds. In care of the Southern Ocean: an archaeological and historical survey of the Auckland Islands. Auckland: New Zealand Archaeological Association.

[PLV104C16] DOC. 1998 Management Strategy Subantarctic Islands 1998–2008, Southland Conservancy Conservation Management Planning Series Number 10.

[PLV104C17] DOC. 2008 Abel Tasman National Park Management Plan. Management Plan Series Nelson: New Zealand Department of Conservation.

[PLV104C18] Egerton R, Burgess S, Petchey P, Dingwell PR 2009 The Auckland Islands shipwreck era. In: Dingwall PR, Jones KL, Egerton R, eds. In care of the Southern Ocean: an archaeological and historical survey of the Auckland Islands. Auckland: New Zealand Archaeological Association.

[PLV104C19] Gillson L, Ekblom A, Willis KJ, Froyd C 2008 Holocene palaeo-invasions: the link between pattern, process and scale in invasion ecology? Landscape Ecology 23:757–769. 10.1007/s10980-008-9243-6

[PLV104C20] Godley EJ 1965 Notes on the vegetation of the Auckland Islands. Proceedings of the New Zealand Ecological Society 12:69–72.

[PLV104C21] Godley EJ 1972 Does planting achieve its purpose? Forest and Bird Journal 185:25–26.

[PLV104C22] Hawke DJ, Newman J 2007 Carbon-13 and nitrogen-15 enrichment in coastal forest foliage from nutrient-poor and seabird-enriched sites in southern New Zealand. New Zealand Journal of Botany 45:309–315. 10.1080/00288250709509719

[PLV104C23] Hay CH, Warham J, Fineran BA 2004 The vegetation of The Snares, islands south of New Zealand, mapped and discussed. New Zealand Journal of Botany 42:861–872. 10.1080/0028825X.2004.9512935

[PLV104C24] Hogg AG, Hua Q, Blackwell PG, Niu M, Buck CE, Guilderson TP, Heaton TJ, Palmer JG, Reimer PJ, Reimer RW, Turney CSM, Zimmerman SRH 2013 SHCal13 Southern Hemisphere calibration, 0–50,000 Years cal BP. Radiocarbon 55:1889–1903. 10.2458/azu_js_rc.55.16783

[PLV104C25] Hooker JD 1844 The Botany of the Antarctic Voyage of H.M. Discovery Ships ‘Erebus’ and ‘Terror’ in the years 1839–1843, under the Command of Captain Sir James Clark Ross. Vol. 1 Flora Antarctica. Part 1. Botany of Lord Auckland's Group and Campbell's Island. London: Reeve.

[PLV104C26] Johnson PN, Campbell DJ 1975 Vascular plants of the Auckland Islands. New Zealand Journal of Botany 13:665–720. 10.1080/0028825X.1975.10430354

[PLV104C27] Lee WG, Wilson JB, Meurk CD, Kennedy PC 1991 Invasion of the subantarctic Auckland Islands, New Zealand, by the asterad tree *Olearia lyallii* and its interaction with a resident myrtaceous tree *Metrosideros umbellata*. Journal of Biogeography 18:493–508. 10.2307/2845686

[PLV104C28] Martinelli LA, Piccolo MC, Townsend AR, Vitousek PM, Cuevas E, McDowell W, Robertson GP, Santos OC, Treseder K 1999 Nitrogen stable isotopic composition of leaves and soil: tropical versus temperate forests. In: Townsend A, ed. New perspectives on nitrogen cycling in the temperate and tropical Americas. The Netherlands: Springer.

[PLV104C29] McCormick R 1884 Voyages of discovery in the Arctic and Antarctic seas, and round the world, Volume 1. London: Sampson Low, Marston, Seale, and Rivington.

[PLV104C30] McCune B, Grace JB, Urban DL 2002 Analysis of ecological communities. Gleneden Beach, OR: MjM Software Design.

[PLV104C31] McGlone M, Wilmshurst J, Meurk C 2007 Climate, fire, farming and the recent vegetation history of subantarctic Campbell Island. Earth and Environmental Science Transactions of the Royal Society of Edinburgh 98:71–84.

[PLV104C32] McGlone MS 2002 The late Quaternary peat, vegetation and climate history of the Southern Oceanic Islands of New Zealand. Quaternary Science Reviews 21:683–707. 10.1016/S0277-3791(01)00044-0

[PLV104C33] McGlone MS, Moar NT 1997 Pollen-vegetation relationships on the subantarctic Auckland Islands, New Zealand. Review of Palaeobotany and Palynology 96:317–338. 10.1016/S0034-6667(96)00058-9

[PLV104C34] McGlone MS, Wilmshurst JM, Wiser SK 2000 Lateglacial and Holocene vegetation and climatic change on Auckland Island, subantarctic New Zealand. The Holocene 10:719–728. 10.1191/09596830094962

[PLV104C35] Meurk CD, Foggo MN, Thomson BM, Bathurst ETJ, Crompton MB 1994 Ion-rich precipitation and vegetation pattern on subantarctic Campbell Island. Arctic and Alpine Research 26:281–289. 10.2307/1551940

[PLV104C36] Moar NT, Wilmshurst JM, McGlone MS 2011 Standardizing names applied to pollen and spores in New Zealand Quaternary palynology. New Zealand Journal of Botany 49:201–229. 10.1080/0028825X.2010.526617

[PLV104C37] Moore PD, Webb JA, Collinson ME 1991 Pollen analysis. Oxford, UK: Blackwell Scientific.

[PLV104C38] Musgrave T 1866 Castaway on the Auckland Islands. London: Lockwood and Co.

[PLV104C39] Petit RJ 2004 Biological invasions at the gene level. Diversity and Distributions 10:159–165. 10.1111/j.1366-9516.2004.00084.x

[PLV104C40] Prickett N 2009 Sealing in the Auckland Islands. In: Dingwall PR, Jones KL, Egerton R, eds. In care of the Southern Ocean: an archaeological and historical survey of the Auckland Islands. Auckland: New Zealand Archaeological Association.

[PLV104C41] Ramsey CB 2008 Radiocarbon dating: revolutions in understanding. Archaeometry 50:249–275. 10.1111/j.1475-4754.2008.00394.x

[PLV104C42] Sawyer J, McFadgen B, Hughes P 2003 Karaka (Corynocarpus laevigatus J.R. et G. Forst.) in Wellington Conservancy (excluding Chatham Islands). DOC Internal Science Series Wellington: New Zealand Department of Conservation.

[PLV104C43] Schofield JE, Edwards KJ, Erlendsson E, Ledger PM 2013 Palynology supports ‘Old Norse’ introductions to the flora of Greenland. Journal of Biogeography 40:1119–1130. 10.1111/jbi.12067

[PLV104C44] Simberloff D, Martin J-L, Genovesi P, Maris V, Wardle DA, Aronson J, Courchamp F, Galil B, García-Berthou E, Pascal M, Pyšek P, Sousa R, Tabacchi E, Vilà M 2013 Impacts of biological invasions: what's what and the way forward. Trends in Ecology and Evolution 28:58–66. 10.1016/j.tree.2012.07.013 22889499

[PLV104C45] Smith I 2002 The New Zealand Sealing Industry. Wellington: Department of Conservation.

[PLV104C46] van Leeuwen JF, Schäfer H, Van der Knaap W, Rittenour T, Björck S, Ammann B 2005 Native or introduced? Fossil pollen and spores may say. An example from the Azores Islands. Neobiota 6:27–34.

[PLV104C47] van Leeuwen JFN, Froyd CA, van der Knaap WO, Coffey EE, Tye A, Willis KJ 2008 Fossil pollen as a guide to conservation in the Galapagos. Science 322:1206 10.1126/science.1163454 19023075

[PLV104C48] Vitousek PM 1988 Diversity and biological invasions of oceanic islands. Biodiversity 20:181–189.

[PLV104C49] Walls G 2009 Picking up the plant trail: botanical evidence of people in the Auckland Islands. In: Dingwall PR, Jones KL, Egerton R, eds. In care of the Southern Ocean. Auckland: New Zealand Archaeological Association.

[PLV104C50] Wang Y, Naumann U, Wright ST, Warton DI 2012 mvabund–an R package for model-based analysis of multivariate abundance data. Methods in Ecology and Evolution 3:471–474. 10.1111/j.2041-210X.2012.00190.x

[PLV104C51] Wardle P 1971 Biological Flora of New Zealand 6. *Metrosideros umbellata* Cav. [Syn. M. lucida (Forst.f.) A. Rich.] (Myrtaceae) Southern Rata. New Zealand Journal of Botany 9:645–671. 10.1080/0028825X.1971.10430227

[PLV104C52] Webb D 1985 What are the criteria for presuming native status? Watsonia 15:231–236.

[PLV104C53] Webber BL, Scott JK 2012 Rapid global change: implications for defining natives and aliens. Global Ecology and Biogeography 21:305–311. 10.1111/j.1466-8238.2011.00684.x

[PLV104C54] Whitehead AL, Lyver POB, Jones CJ, Bellingham PJ, MacLeod CJ, Coleman M, Karl BJ, Drew K, Pairman D, Gormley AM, Duncan RP 2014 Establishing accurate baseline estimates of breeding populations of a burrowing seabird, the grey-faced petrel (*Pterodroma macroptera gouldi*) in New Zealand. Biological Conservation 169:109–116. 10.1016/j.biocon.2013.11.002

[PLV104C55] Whitlock C, Larsen C 2001 Charcoal as a Fire Proxy. In: Smol JP, Birks HJB, Last WM, eds. Tracking environmental change using lake sediments: volume 3 terrestrial, algal, and siliceous indicators. Dordrecht: Kluwer Academic Publishers.

[PLV104C56] Whittaker RJ, Fernández-Palacios JM 2007 Island biogeography: ecology, evolution and conservation. Oxford, UK: Oxford University Press.

[PLV104C57] Willis KJ, Birks HJB 2006 What is natural? The need for a long-term perspective in biodiversity conservation. Science 314:1261–1265. 10.1126/science.1122667 17124315

[PLV104C58] Wilmshurst JM, Bestic KL, Meurk CD, McGlone MS 2004 Recent spread of Dracophyllum scrub on subantarctic Campbell Island, New Zealand: climatic or anthropogenic origins? Journal of Biogeography 31:401–413. 10.1046/j.0305-0270.2003.01029.x

[PLV104C59] Wilson HD 1987 Vegetation of Stewart Island, New Zealand. Department of Scientific and Industrial Research.

