# Supplementary material for: Long-term ecology resolves the timing, region of origin and process of establishment for a disputed alien tree
Source: AoB Plants. 2015 Aug 26;7:plv104. doi: 10.1093/aobpla/plv104 (PMC4612295; doi:10.1093/aobpla/plv104)
Supplement: Additional Information [file supp_7_plv104_index.html]

Long-term ecology resolves the timing, region of origin, and process of establishment for a disputed alien tree — Long-term ecology resolves the timing, region of origin and process of establishment for a disputed alien tree — Additional Information 

# Long-term ecology resolves the timing, region of origin and process of establishment for a disputed alien tree

## Additional Information

Additional Information

- Additional Information - Docx file
