# Supplementary material for: Long-term ecology resolves the timing, region of origin and process of establishment for a disputed alien tree
Source: AoB Plants. 2015 Aug 26;7:plv104. doi: 10.1093/aobpla/plv104 (PMC4612295; doi:10.1093/aobpla/plv104)
Supplement: Additional Information [file supp_plv104_plv104supp.docx]

**Supporting Information**

**Table of contents**

1. **Location and vegetation cover of coring sites**

Fig 1. Coring site Ewing Island

Fig. 2. Coring sites Erebus Cove

**2. Modern pollen percentages under *Olearia lyalli* dominated canopy**

Table 1. Pollen percentages from surface samples

**3. Microphotographs of pollen grains**

Fig.3. Microphotographs of reference and fossil pollen.

**4. Age depth model for Ewing Island core**

Table 2. Calibrated years AD for each depth sampled for pollen

**5. Age depth model for inland Erebus Cove core**

1. **Location and vegetation cover of coring sites**

**
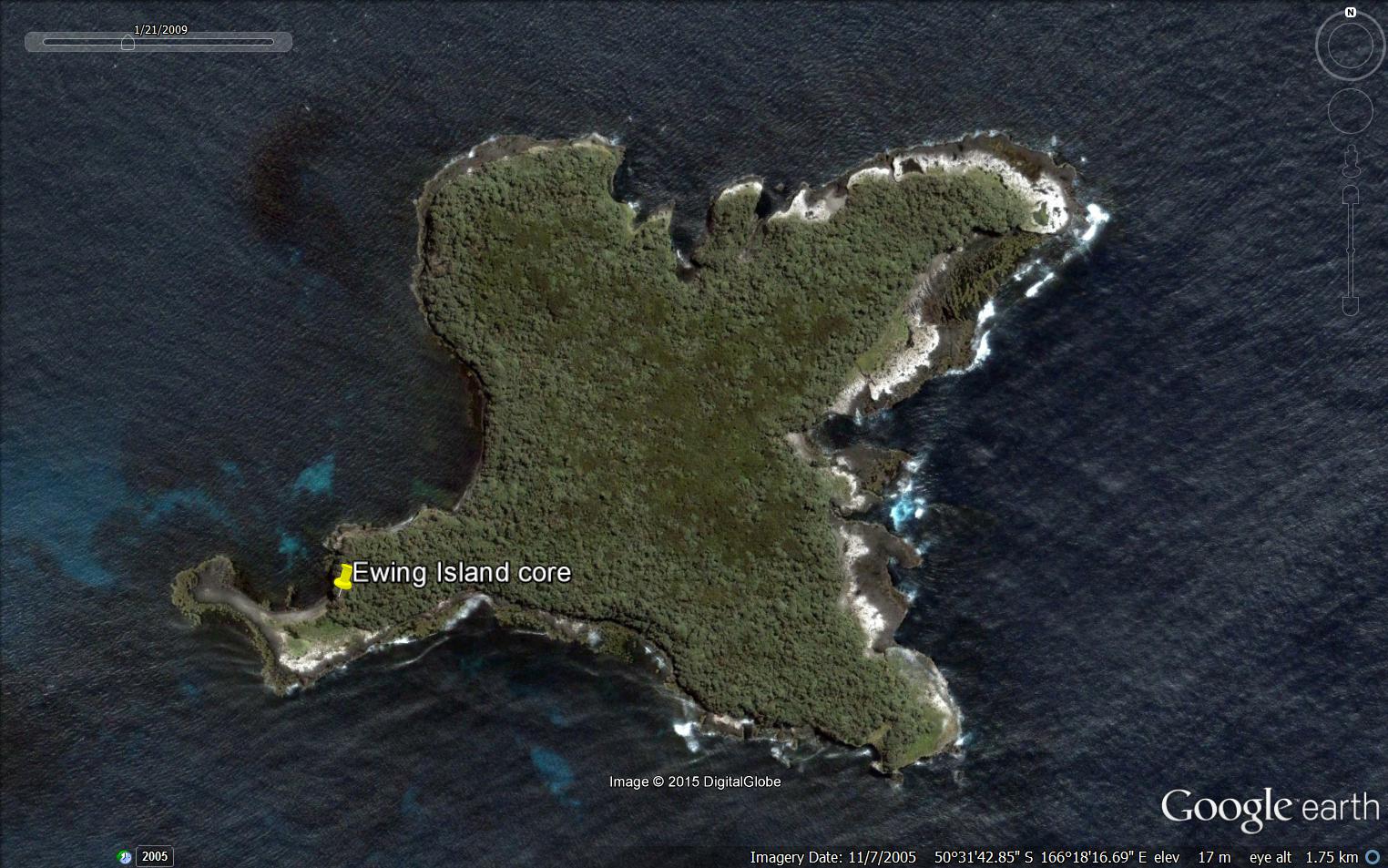
**Fig. 1. Google Earth image (2009) of Ewing Island, showing coastal fringe of *O. lyallii* and central area of dense Metrosideros umbellata forest with coring site adjacent to safe boat landing beach.


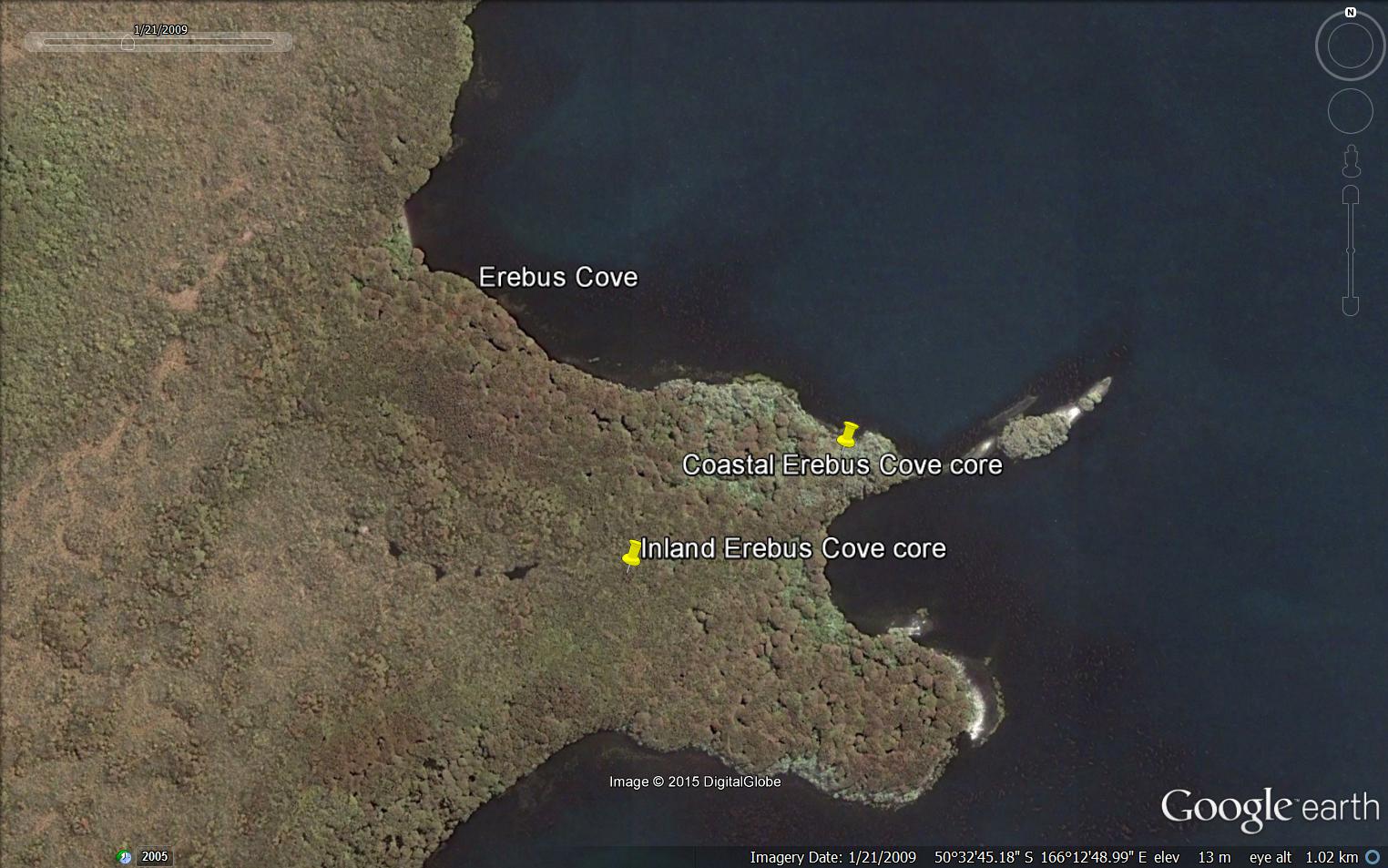
Fig. 2 Google Earth image (2009) of Erebus Cove, Port Ross region of northern Auckland Islands showing locations of coastal *Olearia* Erebus Cove core (X08/22) and Inland *Metrosideros* Erebus Cove core (X08/23). Note the strong contrast between the open whitish-green canopy of *O. lyallii* and the closed reddish-green canopy of *Metrosideros* *umbellata*. Bog communities shown as pale brown vegetation with scattered islands of trees.

**2. Modern pollen percentages under *Olearia lyalli* dominated canopy**

Table 1. Pollen percentages from surface samples taken under *Olearia lyallii* dominated forest canopy on Ewing Island and Snares Island. *Long distance dispersed pollen from southern New Zealand.

| Pollen type | Ewing Island | Snares Island |
| --- | --- | --- |
| *Olearia lyallii* | 83.4 | 50.8 |
| Ground fern spores | 16.7 | 3.8 |
| *Callitriche antarctica* | 7.7 | 0 |
| *Asplenium obfuscatum* | 5.4 | 0 |
| Poaceae | 5.1 | 31.5 |
| *Polystichum vestitum* | 1.5 | 0 |
| **Prumnopitys taxifolia* | 1.5 | 0 |
| **Fuscospora* | 1.5 | 0 |
| *Cyperaceae* | 1.3 | 0 |
| *Anisotome* | 1.3 | 0.8 |
| *Metrosideros umbellata* | 0.9 | 0 |
| *Pinaceae | 0.8 | 0 |
| *Histiopteris incisa* | 0.7 | 0 |
| *Stilbocarpa polaris* | 0.4 | 1.5 |
| **Phyllocladus* | 0.3 | 0 |
| **Eucalyptus* | 0.3 | 0 |

**3. Microphotographs of pollen grains**

Fig. 3. Microphotographs of reference grains (a) *Pleurophyllum hookerii* type; (b) *P. speciosum;* (c) *Olearia lyallii*; and fossil grain (d) *O. lyallii* from Ewing Island core (10 cm depth). Polar axis: a, c, d: 29-33 µm; b: 31-34 µm ([Moar, 1993](#_ENREF_2)).


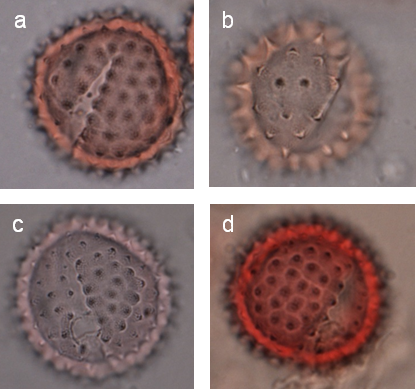


**4. Age depth model for Ewing Island core (X13/84)**

Table 2. Calibrated ages for pollen sample depths in the Ewing Island core, in years Before Present (BP; where present =1950) and calibrated years AD using the P_sequence option in OxCal ([Ramsey, 2008](#_ENREF_3)) using the SHCal13 calibration dataset ([Hogg et al., 2013](#_ENREF_1)), and depths where *Olearia* *lyallii* pollen first occurs and then exceeds 5% of the pollen sum.

|  |  |  |
| --- | --- | --- |
|  |  |  |
|  |  |  |
|  |  |  |
|  |  |  |
|  |  |  |
|  |  |  |
|  |  |  |
|  |  |  |
|  |  |  |
|  |  |  |
|  |  |  |
|  |  |  |
|  |  |  |
|  |  |  |
|  |  |  |
|  |  |  |
|  |  |  |
|  |  |  |
|  |  |  |
|  |  |  |
|  |  |  |
|  |  |  |
|  |  |  |
|  |  |  |
|  |  |  |
|  |  |  |
|  |  |  |
|  |  |  |
|  |  |  |
|  |  |  |

| **Depth (cm) in core** | **Cal yrs BP** | **Cal yrs AD from 2013 time of collection** | **Olearia** |
| --- | --- | --- | --- |
| 2 | -57.9 | 2008 |  |
| 4 | -52.7 | 2003 |  |
| 6 | -47.6 | 1998 |  |
| 8 | -42.5 | 1993 |  |
| 10 | -37.3 | 1987 |  |
| 12 | -23.1 | 1973 |  |
| 14 | -5.9 | 1956 |  |
| 16 | 11.2 | 1939 |  |
| 18 | 28.4 | 1922 |  |
| 20 | 45.6 | 1904 |  |
| **24** | **80** | **1870** | *Olearia lyallii* >5% |
| 28 | 119.8 | 1830 |  |
| **32** | **163** | **1787** | First trace of *Olearia lyallii* |
| 36 | 206.1 | 1744 |  |
| 40 | 249.2 | 1701 |  |
| 44 | 292.4 | 1658 |  |
| 48 | 335.2 | 1615 |  |
| 52 | 377.8 | 1572 |  |
| 56 | 420.5 | 1530 |  |
| 60 | 463.1 | 1487 |  |
| 64 | 505.7 | 1444 |  |
| 68 | 548.4 | 1402 |  |
| 72 | 591 | 1359 |  |
| 76 | 629.2 | 1321 |  |
| 80 | 636.3 | 1314 |  |
| 84 | 814.6 | 1135 |  |
| 88 | 1017.3 | 933 |  |
| 92 | 1220 | 730 |  |
| 96 | 1422.7 | 527 |  |
| 99 | 1574.8 | 375 |  |


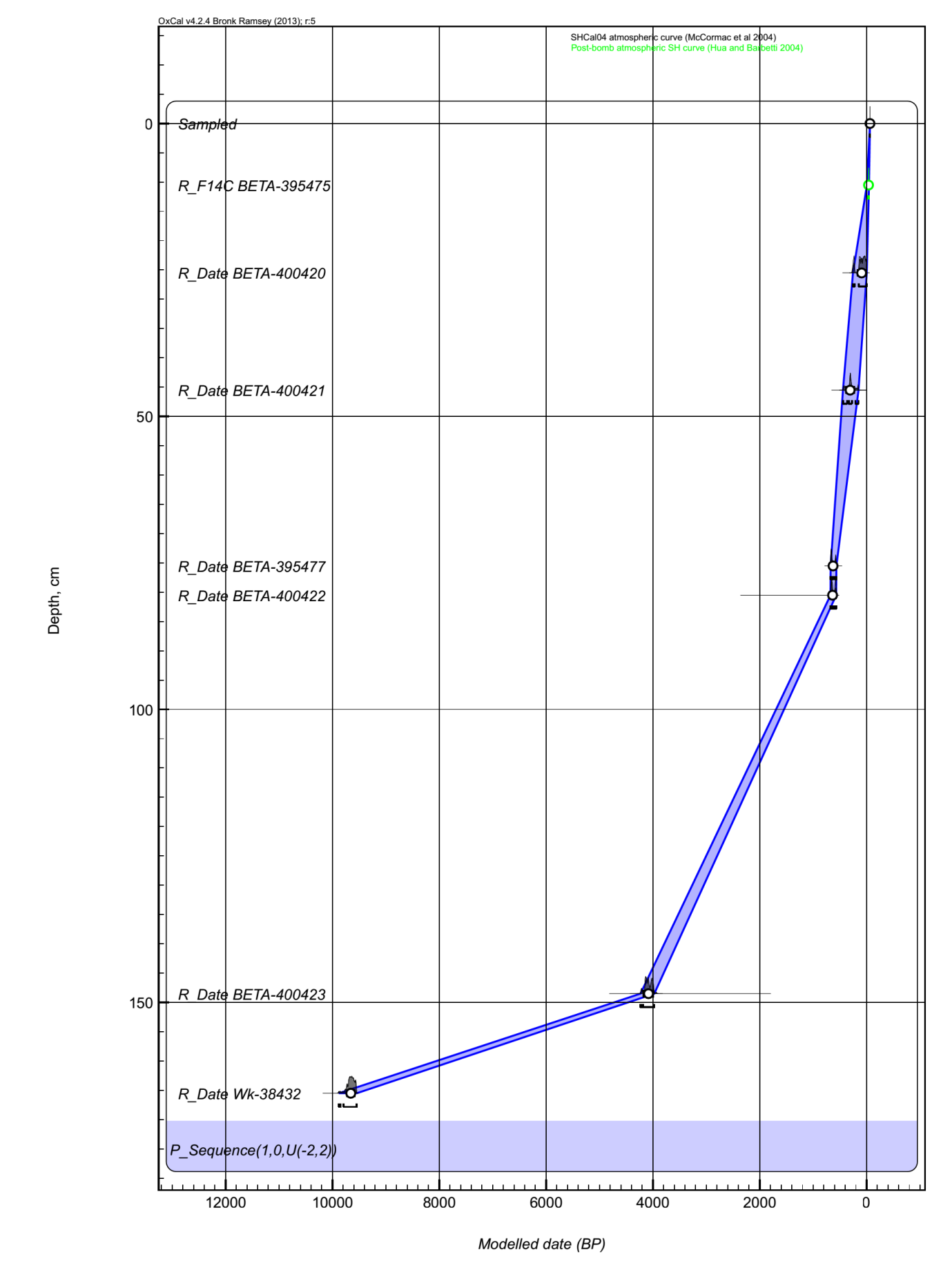
Fig 4. Age depth model for Ewing Island core

**5. Age depth model for coastal Erebus Cove core (X08/22)**

Table 3. Linear interpolation between calibrated radiocarbon ages in coastal Erebus Cove core, and and depths where *Olearia* *lyallii* pollen first occurs and then exceeds 5% of the pollen sum.

5 cm = 1997

50 cm = 1815-1830

Using 1815-1997: Predicted age = (-4.0444*depth) + 2017.2

Using 1830-1997: Predicted age = (-3.7111x depth) + 2015.6

| Depth (cm) | Age cal yr AD | Event |
| --- | --- | --- |
| 0 | 2017-2016 |  |
| 1 | 2013-2012 |  |
| 2 | 2009-2008 |  |
| 4 | 2001 |  |
| 6 | 1993 |  |
| 8 | 1985-1986 |  |
| 10 | 1977-1978 |  |
| 12 | 1969-1971 |  |
| 14 | 1961-1964 |  |
| 16 | 1952-1956 |  |
| 18 | 1944-1949 |  |
| 20 | 1936-1941 |  |
| 22 | 1928-1934 |  |
| 24 | 1920-1927 |  |
| 26 | 1912-1919 |  |
| 28 | 1904-1912 |  |
| 30 | 1896-1904 |  |
| 32 | 1888-1897 |  |
| 34 | 1880-1889 |  |
| 36 | 1872-1882 |  |
| 38 | 1864-1875 |  |
| 40 | 1855-1867 |  |
| 42 | **1847-1860** | *Olearia* pollen increases |
| 44 | 1839-1852 |  |
| 46 | 1831-1845 |  |
| 48 | **1823-1837** | First trace *Olearia* pollen |
| 50 | 1815-1830 |  |

**Citations**

**Hogg AG, Hua Q, Blackwell PG, Niu M, Buck CE, Guilderson TP, Heaton TJ, Palmer JG, Reimer PJ, Reimer RW, Turney CSM, Zimmerman SRH.** **2013**. SHCal13 Southern Hemisphere calibration, 0–50,000 Years cal BP. *Radiocarbon,* **55**: 1889-1903.

**Moar NT.** **1993**. *Pollen grains of New Zealand dicotyledonous plants,* Lincoln, New Zealand, Manaaki Whenua Press.

**Ramsey CB.** **2008**. Radiocarbon dating: revolutions in understanding. *Archaeometry,* **50**: 249-275.
